# The Cedar Project: Racism and its impacts on health and wellbeing among young Indigenous people who use drugs in Prince George and Vancouver, BC

**DOI:** 10.1371/journal.pgph.0001914

**Published:** 2023-08-30

**Authors:** Richa Sharma, Sherri Pooyak, Vicky Thomas, David S. Zamar, Kate Jongbloed, Margo E. Pearce, April Mazzuca, Chenoa Cassidy-Mathews, Riley N. Bizzotto, Ghazal Jafari, Kukpi7 Wayne M. Christian, Mary Teegee, Martin T. Schechter, Patricia M. Spittal

**Affiliations:** 1 University of British Columbia, Vancouver, Canada; 2 The Cedar Project Partnership and Aboriginal HIV/AIDS Community-Based Research Collaborative Centre (AHA Centre), Vancouver, Canada; 3 The Cedar Project, Vancouver, Canada; 4 Children’s Hospital Research Institute, University of British Columbia, Vancouver, BC, Canada; 5 School of Population and Public Health, Faculty of Medicine, University of British Columbia, Vancouver, Canada; 6 McGill University Department of Psychology, The Cedar Project, Vancouver, Canada; 7 The Cedar Project Partnership and Splatsin te Secwepemc, Vancouver, Canada; 8 The Cedar Project Partnership and Carrier Sekani Family Services, Vancouver, Canada; University of the Southern Caribbean, TRINIDAD AND TOBAGO

## Abstract

Racism continues to drive health disparities between Indigenous and non-Indigenous peoples in Canada. This study focuses on racism experienced by young Indigenous people who have used drugs in British Columbia (BC), and predictors of interpersonal racism. Cedar Project is a community-governed cohort study involving young Indigenous people who use drugs in Vancouver and Prince George, BC. This cross-sectional study included data collected between August 2015-October 2016. The Measure of Indigenous Racism Experiences (MIRE) scale was used to assess experiences of interpersonal racism across 9 unique settings on a 5-point Likert scale, collapsing responses into three categories (none/low/high). Multinomial logistic regression models were used to examine associations between key variables and interpersonal racism. Among 321 participants, 79% (n = 255) experienced racism in at least one setting. Thirty two percent (n = 102) experienced high interpersonal racism from police, governmental agencies (child ‘welfare’, health personnel), and in public settings. Ever having a child apprehended (AOR:2.76, 95%CI:1.14–6.65), probable post-traumatic stress (AOR:2.64; 95%CI:1.08–6.46), trying to quit substances (AOR:3.69; 95%CI:1.04–13.06), leaving emergency room without receiving treatment (AOR:3.05; 95%CI:1.22–7.64), and having a traditional language spoken at home while growing up (AOR:2.86; 95%CI:1.90–6.90) were associated with high interpersonal racism. Among women, experiencing high interpersonal racism was more likely if they lived in Prince George (AOR:3.94; 95%CI:1.07–14.50), ever had a child apprehended (AOR:5.09; 95%CI:1.50–17.30), and had probable post-traumatic stress (AOR:5.21; 95%CI:1.43–18.95). Addressing racism experienced by Indigenous peoples requires immediate structural systemic, and interpersonal anti-racist reforms.

## Introduction

Ongoing settler colonialism is a key determinant of Indigenous peoples’ health and wellness [1–5]. Health impacts of Indigenous-specific racism include reduced access to health services [6–8] and negative physical and mental health outcomes [6, 9–12]. Mounting evidence points to the role of imposed colonial systems and racism in post-traumatic stress disorder (PTSD) and other markers of trauma among Indigenous peoples [13, 14]. Yet, most structures and systems that perpetuate racist harm remain in place. In June 2020, BC initiated a province-wide investigation following allegations of Indigenous-specific racism, leading to a damning report on pervasive Indigenous-specific racism in BC’s healthcare system [6]. Over 84% of 2,780 Indigenous respondents indicated that they had experienced some form of discrimination in health care, and 13% of the 5,440 health worker respondents made racist comments in the survey itself [6]. In September 2020, Joyce Echaquan (Atikamekw Nation) was seeking help for stomach pain at Centre Hospitalier de Lanaudière in Joliette, Quebec [15]. Out of concern for her health and safety, she live-streamed her experience of racism on social media. The recording captured healthcare workers taunting and insulting her, proclaiming, “You’re stupid as hell…” and “She’s good at having sex, more than anything else,” among other derogatory slurs. Ms. Echaquan died soon after. Despite recent attention, the vast majority of racist incidents go unrecorded and unreported due to systemic racism in complaints processes [6, 16].

Since time immemorial, Indigenous peoples have lived in the territories now known as “British Columbia” (BC) [17]. The ancestral territories of BC First Nations stretch across every inch of ‘British Columbia’. BC is also home to other Indigenous people and communities including First Nations, Métis and Inuit peoples and others with Indigenous identity. We use the broad term “Indigenous” within this article to reflect the diversity of Nations and Peoples who call this province home, while also respectfully acknowledging the territorial rights of BC First Nations who are the original occupants of these lands. European visitors arrived in the 1700s armed with the “Doctrine of Discovery,” an oppressive edict from the Catholic Church which claimed that any territory occupied by non-Christians was “empty and uninhabited” (terra nullius). Rather than recognizing existing sovereign Indigenous Nations, the Doctrine justified legal and moral claims to sovereignty over lands for their monarchs. Thus began the imposition of foreign laws, policies, and institutions rooted in white supremacy and beliefs about inferiority of Indigenous peoples, aiming to dispossess Indigenous peoples from their lands, cultures, and family ties [17]. Since 1876, the *Indian Act*, a race-based legislation, has imposed control over every aspect of Indigenous lives, from birth to death, through assimilationist policies, including residential ‘schools’, enforced by the police [18–21]. Racism is foundational to settler colonialism across Canada, contributing to enduring inequities that privilege settler Canadians while oppressing Indigenous peoples [22, 23]. Indigenous-specific racism occurs at multiple levels–structural (laws and policies), systemic (organizations and programs), and services (interpersonal interactions)–each reinforcing the other [24, 25]. While Indigenous peoples, Nations, and ways of being have survived, we must acknowledge that settler society continues to place the burden on Indigenous peoples to survive and resist, rather than enacting transformative change.

Indigenous-specific racism intersects with and exacerbates other forms of discrimination, including laws and policies based on sexism, stigma towards mental illness, and criminalization of drug use. To our knowledge, no epidemiologic studies have examined racism experienced by young Indigenous people who use drugs in BC. This study identifies prevalence and factors associated with experiences of racism to inform policy and service provision. It focuses on three Canadian institutions which continue to cause racist harm to Indigenous peoples and their families: the health system (and emerging mental health and addictions system); child apprehension systems (including residential ‘schools’ and child ‘welfare’ systems); and the criminal justice system (including drug policy). Disaggregated analysis by sex stems from recognition of the gendered nature of settler colonialism and that experiences of racism may be different for Indigenous men and women [6, 26].

## Materials and methods

### Study design

The Cedar Project (Cedar) is an Indigenous-governed cohort initiated in 2003 and was established to explore the lived experiences of HIV/HCV among young Indigenous people who use criminalized substances in BC. Our Indigenous-governance, the Cedar Project Partnership, is an independent body of Indigenous Elders, knowledge keepers and health and social service experts. They govern all aspects of Cedar research and process. Our research offices are located on Lheidli T’enneh (Prince George) and Coast Salish territories (Vancouver). Cedar methods have previously been described in detail [27]. In brief, participants were enrolled, by non-probabilistic sampling, if they self-identified as Indigenous, smoked or injected criminalized substances other than cannabis (e.g., crystal methamphetamine, crack-cocaine, heroin, cocaine, or opioids) at least one month before enrollment, were between 14–30 years of age at baseline, and provided informed consent. We define “Indigenous” as referring to people who are descendants of the First peoples of North America, including Métis, Aboriginal, First Nations, Inuit, and Status and non-Status Indians. Participants returned every 6 months for follow-up. Honoraria were provided. This cross-sectional analysis included 296 participants who completed a follow-up questionnaire between 2015–2016, and the interviewer administered Measure of Indigenous Racism Experiences (MIRE) scale [28].

### Outcome measure

The outcome variable, perceived racial discrimination, was indicated by the interpersonal racism score captured by the MIRE. Interpersonal racism is racism that occurs between individuals. Created in Australia, this is the first validated instrument to address the unique and multiple facets of racism experienced by Indigenous peoples and has been previously adopted in Canadian settings [28, 29]. Participants were asked about experiences of “being treated unfairly because they are Indigenous” across 9 settings on a 5-point Likert scale: “never”, “hardly ever”, “sometimes”, “often”, “very often”. Settings included interactions at work, at home, at school, while doing leisure activities, by the police, by doctors, by staff of government agencies such as the Ministry of Children and Family Development (MCFD), public spaces such as restaurants, shops and stores, and by other people on the street. Experiences of interpersonal racism were collapsed into 3 levels: none (all a-i item responses ’never’), low (average response of ’hardly ever’ for items a-i) and high (average response of ’sometimes’, ’often’, or ’very often’ for items a-i) [28, 30]. A total of 25 (7.8%) participants were missing a response for more than 2 items and excluded from regression analyses. Cronbach’s α was 0.91, indicating good internal consistency.

### Exposure variables

We received direction from our Partnership to examine the relationships between racism and several variables measuring historical and ongoing colonial trauma, substance use, access to services and treatment, HIV/HCV infection, and cultural connection. Examined variables included: age (years), sex (male/female), city (Vancouver/Prince George), sexuality (heterosexual/Two-Spirit), education (<high school/≥high school), relationship status (relationship/single). Participants reported if their parents attended residential schools (no/unsure/yes), and if they had ever (no/yes) been taken into foster care, were sexually abused as a child, had ever slept outside >3 nights in a row, ever went to jail or detention, ever physically assaulted, ever sexually assaulted, ever attempted suicide, ever overdosed, ever injected drugs, ever tried to quit using alcohol and drugs, ever been paid for sex, ever been denied shelter, ever been denied any services, ever tried to access treatment but were unable to, ever left the emergency room without receiving treatment, and had probable post-traumatic stress, defined as scoring above 30 on the PTSD Checklist civilian version (PCL-C)(25). Finally, we measured impact of cultural connections, including whether participants (rarely-never/often-always) participated in traditional ceremonies, had a traditional language spoken at home growing up, and their family lived by traditional culture.

### Statistical analysis

Responses to the MIRE were analyzed as simple univariate proportions. Associations with other factors were examined via Chi-square test or Fisher’s exact tests of association. Multinomial logistic regression models identified factors associated with severity (none/low/high) of interpersonal racism. Unadjusted and adjusted odds ratios with corresponding 95% confidence intervals were calculated using cumulative logit models with severity levels as ordinal categorical response variables. Variables significant at *p*<0.10 were included in a multivariable model adjusted for age, location, and gender. Gender-stratified results are also presented. Variables with cell counts less than five were excluded from regression analyses. Available case-analysis was undertaken for all participants with outcome data (n = 296) and no imputation was conducted for missing data.

### Ethical considerations

We followed the Tri-Council Policy Statement: Ethical Conduct for Research Involving Humans, with attention to chapter 9, pertaining to research involving Indigenous peoples [31]. Since Cedar’s inception, it has been governed by the Cedar Project Partnership, a body of Indigenous leaders, Elders, community members and health and social services experts. UBC-Providence Health Care Research Ethics Board approved the study.

At enrolment, all participants met with an Indigenous Study Coordinator who described the study, confirmed eligibility, and requested informed consented after explaining potential benefits and harms associated with participation. After the age of 14, minors may consent for themselves regarding their own medical care, therefore consent is not sought from parents or guardians. All participants receive a copy of the consent form and staff verbally review the full consent form with participants. The consenting process may take from 10 to 60 minutes depending on the participant and the number of questions for staff about the study. It is vital for all staff to review the consent form with participants outlining participant rights throughout the research process and explaining what will occur at every stage. Nothing should come as a surprise to participants throughout the duration of their involvement with Cedar.

## Results

Among 321 participants who completed the MIRE (Table 1), 79% (n = 255) reported experiencing racism in more than one setting. Almost 40% (n = 125) reported experiencing racism across 6–9 settings. Participants reported being treated unfairly most frequently by police and security personnel (45%), in public spaces (42%), by staff of agencies like MCFD (37%), by people on the streets (37%), and by doctors or staff at hospitals (34%).

**Table 1:** Number of settings in which racism was reported (n = 321).

Number of settings	0	1 to 2	3 to 5	6 to 9	NA
**Count**	54 (17.5%)	54 (17.5%)	76 (24.6%)	125 (40.4%)	12

More than half of participants (58%; n = 171) were women, and 57% (n = 172) enrolled in Prince George (Table 2). Median age was 33 years (IQR:30–37). Thirty-eight percent (n = 117) said their traditional language was spoken at home always/often while growing up, and 30% (n = 95) said their family lived by traditional culture. However, 47% (n = 140) had a parent who attended residential school, 75% (n = 222) had been apprehended from their biological parents, and 49% (n = 144) reported having had their own child apprehended.

**Table 2:** Baseline characteristics of young Indigenous people who reported experiencing no racism (n = 49), low racism (n = 145), and high racism (n = 102).

Characteristic	Row total		No racism		Low racism		High racism		*p* *
**Demographic**	n	%	n	%	n	%	n	%	
Gender									
Male	125	42.3%	27	21.6%	66	52.8%	32	25.6%	**0.012**
Female	171	57.8%	22	12.9%	79	46.2%	70	40.9%	
City									
Vancouver	124	41.9%	30	24.2%	61	49.2%	33	26.6%	**0.003**
Prince George	172	58.1%	19	11.0%	84	48.8%	69	40.1%	
Age in years, median (IQR)			33.59	(30.1–37.7)	***33*.*2***	(29.7–36.8)	32.1	(29.9–36.5)	0.479
Sexual Identity									
Straight	254	83.1%	42	16.5%	125	49.2%	87	34.3%	0.935
Two-Spirit	41	13.9%	6	14.6%	20	48.8%	15	36.6%	
Education level									
Less than high school	243	83.2%	38	15.6***%***	***119***	49.0%	86	35.4%	0.562
High school or higher	49	16.8%	10	20.4%	25	51.0%	14	28.6%	
Relationship status									
In a relationship	142	49.0%	22	15.5%	76	53.5%	44	31.0%	0.368
Single	148	51.0%	26	17.6%	67	45.3%	55	37.2%	
**Historical & Ongoing colonial trauma**									
Either parent attended residential school									
No	86	29.3%	13	15.1%	43	50.0%	30	34.9%	0.142
Unsure	68	23.1%	16	23.5%	36	52.9%	16	23.5%	
Yes	140	47.6%	19	13.6%	65	46.4%	56	40.0%	
Ever removed from biological parents									
No	74	25.0%	16	21.6%	***34***	45.9%	24	32.4%	0.400
Yes	222	75.0%	33	14.9%	111	50.0%	78	35.1%	
Childhood sexual abuse (≤ 15 yr)									
No	146	50.7%	29	19.9%	79	54.1%	38	26.0%	**0.009**
Yes	142	49.3%	19	13.4%	62	43.7%	61	43.0%	
Ever had a child apprehended									
No	152	51.4%	33	21.7%	76	50.0%	43	28.3%	**0.014**
Yes	144	48.6%	16	11.1%	69	47.9%	59	41.0%	
Ever slept out > 3 nights in a row									
No	27	9.1%	7	25.9%	13	48.1%	7	25.9%	0.332
Yes	269	90.9%	42	15.6%	132	49.1%	95	35.3%	
Ever been in detention/prison									
No	61	20.6%	8	13.1%	35	57.4%	18	29.5%	0.333
Yes	235	79.4%	41	17.4%	***110***	46.8%	84	35.7%	
Ever been physically assaulted									
No	97	32.8%	18	18.6%	51	52.6%	28	28.9%	0.361
Yes	199	67.2%	31	15.6%	94	47.2%	74	37.2%	
Ever attempted suicide									
No	168	59.2%	35	20.8%	***85***	50.6%	48	28.6%	**0.027**
Yes	116	40.8%	13	11.2%	55	47.4%	48	41.4%	
Ever sexually assaulted									
No	118	39.9%	25	21.2%	62	52.5%	31	26.3%	**0.032**
Yes	178	60.1%	24	13.5%	83	46.6%	71	39.9%	
Ever been forced to have sex with a cop									
No	285	96.9%	46	16.1%	142	49.8%	97	34.0%	**0.040**
Yes	9	3.1%	3	33.3%	1	11.1%	5	55.6%	
Ever been in an incident where police used physical force against you									
No	170	57.4%	30	17.6%	87	51.2%	53	31.2%	0.381
Yes	126	42.6%	19	15.1%	58	46.0%	49	38.9%	
Ever been stopped by the police									
No	35	11.8%	4	11.4%	20	57.1%	11	31.4%	0.594
Yes	261	88.2%	45	17.2%	125	47.9%	91	34.9%	
Ever been paid for sex									
No	138	46.6%	24	17.4%	***73***	52.9%	41	29.7%	0.271
Yes	158	53.4%	25	15.8%	72	45.6%	61	38.6%	
Probable Post-traumatic stress									
PCL Score Below 30	93	31.8%	25	26.9%	48	51.6%	20	21.5%	**<0.001**
PCL Score 30 or Above	199	68.2%	23	11.6%	94	47.2%	82	41.2%	
**Substance use**									
Ever overdosed									
No	182	61.5%	33	18.1%	95	52.2%	54	29.7%	**0.088**
Yes	114	38.5%	16	14.0%	50	43.9%	48	42.1%	
Ever injected									
No	85	28.7%	15	17.6%	46	54.1%	24	28.2%	0.356
Yes	211	71.3%	34	16.1%	99	46.9%	78	37.0%	
**Access to services and treatment**									
Ever been denied shelter									
No	125	42.2%	23	18.4%	64	51.2%	38	30.4%	0.428
Yes	171	57.8%	26	15.2%	81	47.4%	64	37.4%	
Ever tried to access treatment but unable to									
No	114	41.3%	21	18.4%	61	53.5%	32	28.1%	0.186
Yes	162	58.7%	28	17.3%	64	39.5%	70	43.2%	
Ever been denied services									
No	163	55.1%	28	17.2%	96	58.9%	39	23.9%	**<0.001**
Yes	133	44.9%	21	15.8%	49	36.8%	63	47.4%	
Ever tried to quit using alcohol or drugs									
No	41	13.9%	13	31.7%	20	48.8%	8	19.5%	**0.008**
Yes	255	86.1%	36	14.1%	125	49.0%	94	36.9%	
Ever gone to ER or clinic and left before receiving treatment									
No	195	65.9%	40	20.5%	106	54.4%	49	25.1%	**<0.001**
Yes	101	34.1%	9	8.9%	39	38.6%	53	52.5%	
**HIV/HCV infection**									
HIV+									
No	227	81.9%	38	16.7%	***111***	48.9%	78	34.4%	0.849
Yes	50	18.1%	10	20.0%	24	***48*.*0%***	16	32.0%	
HCV+									
No	132	47.0%	22	16.7%	69	52.3%	41	31.1%	0.580
Yes	149	53.0%	26	17.4%	69	46.3%	54	36.2%	
**Cultural Connections**									
Live by traditional culture									
Never/Rarely	251	86.3%	45	17.9%	124	49.4%	82	32.7%	0.254
Always/Often	40	13.7%	4	10.0%	18	45.0%	18	45.0%	
Participated in traditional ceremonies									
No	244	83.0%	43	17.6%	119	48.8%	82	33.6%	0.597
Yes	50	17.0%	6	12.0%	25	50.0%	19	38.0%	
Traditional language spoken at home									
Never/Rarely	175	59.9%	38	21.7%	80	***45*.*7%***	57	32.6%	**0.022**
Always/Often	117	40.1%	11	9.4%	62	53.0%	44	37.6%	
Do you know how to speak your language									
No	180	61.0%	30	16.7%	87	48.3%	63	35.0%	0.986
A bit	94	31.9%	15	16.0%	48	51.1%	31	33.0%	
Yes	21	7.1%	4	19.0%	10	47.6%	7	33.3%	
How often do you speak your traditional language today									
Never/Rarely	274	92.1%	46	16.8%	135	49.3%	93	33.9%	0.954
Always/Often	21	7.1%	3	14.3%	10	47.6%	8	38.1%	
How much does your family live by traditional culture									
Never/Rarely	136	58.9%	27	19.9%	62	45.6%	47	34.6%	0.243
Always/Often	95	41.1%	11	11.6%	49	51.6%	35	36.8%	

*Chi-square test of association. Fisher’s test was used if a cell count was less than 5.

In multivariable analysis (Table 3), factors associated with ***experiencing*** high levels of racism included having had a child apprehended, living with probable post-traumatic stress, having tried to quit alcohol and drug use, having left ER without receiving treatment, and having had a traditional language spoken at home while growing up.

**Table 3:** Bivariate and multivariate multinomial logistic regression analysis of factors associated with racism among all participants (n = 296).

Characteristic	UOR (95% CI)		AOR (95% CI)	
Socio-demographic	Low racism	High racism	Low racism	High racism
Gender, female	1.47 (0.77–2.82)	**2.68 (1.33–5.41)**	0.87 (0.37–2.02)	1.62(0.62–4.21)
City, Prince George	**2.17 (1.12–4.22)**	**3.30 (1.63–6.71)**	1.40 (0.64–3.05)	1.27 (0.53–3.09)
Age	**0.99 (0.93–1.05)**	**0.97 (0.91–1.04)**	1.00 (0.93–1.08)	0.97 (0.90–1.06)
Identify as Two-Spirit	1.12 (0.42–2.98)	1.21 (0.44–3.33)		
Education level, high school or more	0.80 (0.35–1.81)	0.62 (0.25–1.52)		
Relationship status, single	0.75 (0.39–1.44)	1.06 (0.53–2.11)		
**Historical and ongoing colonial trauma**				
Either parents attended residential school				
Unsure	0.68 (0.28–1.60)	0.43 (0.17–1.12)		
Yes	1.03 (0.46–2.31)	1.28 (0.56–2.94)		
Removed from biological parents	1.58 (0.78–3.22)	1.58 (0.74–3.34)		
Childhood sexual abuse ≤15	1.20 (0.61–2.33)	**2.45 (1.21–4.96)**	0.68 (0.18–2.50)	1.29 (0.29–5.89)
Ever had a child apprehended	1.87 (0.95–3.70)	**2.83 (1.38–5.78)**	1.93 (0.86–4.35)	**2.76 (1.14–6.65)**
Ever slept outside > 3 nights in a row	1.69 (0.63–4.52)	2.26 (0.75–6.86)		
Ever went to jail or detention	0.61 (0.26–1.43)	0.91 (0.37–2.27)		
Ever physically assaulted	1.07 (0.55–2.10)	1.53 (0.74–3.17)		
Ever attempted suicide	1.74 (0.85–3.48)	**2.69 (1.27–5.71)**	1.07 (0.46–2.48)	1.29 (0.51–3.21)
Ever sexually assaulted	1.39 (0.73–2.67)	**2.39 (1.18–4.81)**	1.73 (0.47–6.39)	1.06 (0.22–5.02)
Police ever used physical force against you	1.05 (0.54–2.04)	1.46 (0.73–2.92)		
Ever paid for sex work	0.95 (0.50–1.81)	1.43 (0.72–2.84)		
Probable Post-traumatic stress	**2.13 (1.10–4.14)**	**4.45 (2.11–9.42)**	1.62 (0.75–3.48)	**2.64 (1.08–6.46)**
**Substance Use**				
Ever overdosed	1.08 (0.54–2.16)	1.83 (0.90–3.73)		
Ever injected	0.95 (0.47–1.91)	1.43 (0.67–3.07)		
Ever tried to quit alcohol and drugs	**2.26 (1.02–4.98)**	**4.24 (1.62–11.09)**	1.83 (0.73–4.58)	**3.69 (1.04–13.06)**
**Access to services and treatment**				
Ever been denied shelter	1.12 (0.58–2.14)	1.49 (0.75–2.97)		
Ever denied any services	0.68 (0.35–1.32)	**2.15 (1.08–4.31)**	0.57 (0.27–1.23)	1.55 (0.67–3.59)
Ever tried to access treatment but unable to	1.03 (0.54–1.99)	1.64 (0.81–3.32)		
Ever left ER without receiving treatment	1.64 (0.73–3.68)	**4.81 (2.12–10.93)**	1.07 (0.44–2.57)	**3.05 (1.22–7.64)**
**HIV & HCV infection**				
HIV+	0.82 (0.36–1.87)	0.78 (0.32–1.88)		
HCV+	0.85 (0.44–1.63)	1.11 (0.55–2.24)		
**Cultural Connections**				
Participated in traditional ceremonies	1.51 (0.58–3.92)	1.66 (0.62–4.47)		
Traditional language always/often spoken at home	**2.68 (1.27–5.66)**	**2.67 (1.23–5.80)**	**2.38 (1.06–5.31)**	**2.86 (1.9–6.90)**
Family always/often live by traditional culture	1.94 (0.88–4.30)	1.83 (0.80–4.18)		

Among women (Table 4), 87% (n = 149) reported experiencing racism in more than one setting. In multivariable analysis, living in Prince George, ever having had a child apprehended, and having probable post-traumatic stress remained significantly associated with experiencing high levels of racism.

**Table 4:** Bivariate and multivariate multinomial logistic regression analysis of factors associated with racism among women (n = 187).

Characteristic*	UOR (95% CI)		AOR (95% CI)	
Socio-demographic	Low racism	High racism	Low racism	High racism
City, Prince George	**2.71 (1.02–7.21)**	**5.46 (1.96–15.22)**	2.00 (0.60–6.61)	**3.94 (1.07–14.50)**
Age	**1.02 (0.93–1.12)**	**1.03 (0.94–1.14)**	1.00 (0.90–1.12)	1.01 (0.90–1.14)
Identify as Two-Spirit	0.67 (0.21–2.14)	0.57 (0.17–1.88)		
Relationship status, single	1.93 (0.68–5.51)	**2.81 (0.97–8.12)**	1.26 (0.39–4.03)	1.25 (0.36–4.30)
**Historical and ongoing colonial trauma**				
Either parents attended residential school				
Unsure	0.80 (0.21–3.02)	0.90 (0.22–3.66)		
Yes	0.67 (0.22–2.04)	1.43 (0.45–4.52)		
Removed from biological parents	1.04 (0.36–2.30)	1.27 (0.42–3.77)		
Childhood sexual abuse ≤15	0.90 (0.34–2.36)	2.08 (0.76–5.71)		
Ever had a child apprehended	1.86 (0.72–4.82)	**2.62 (0.98–6.97)**	2.28 (0.77–6.71)	**5.09 (1.50–17.30)**
Ever went to jail or detention	0.54 (0.18–1.61)	0.99 (0.32–3.11)		
Ever physically assaulted	1.19 (0.45–3.13)	1.51 (0.56–4.06)		
Ever attempted suicide	2.38 (0.79–7.18)	**3.39 (1.11–10.31)**	2.45 (0.53–11.32)	1.88 (0.40–8.72)
Ever sexually assaulted	1.11 (0.38–3.21)	1.50 (0.50–4.53)		
Police ever used physical force against you	0.96 (0.36–2.57)	1.17 (0.43–3.14)		
Probable Post-traumatic stress	2.30 (0.87–6.07)	**4.00 (1.44–11.10)**	1.77 (0.56–5.55)	**5.21 (1.43–18.95)**
**Substance Use**				
Ever overdosed	1.24 (0.45–3.40)	1.91 (0.69–5.26)		
Ever injected	0.93 (0.30–2.85)	1.18 (0.37–3.74)		
**Access to services and treatment**				
Ever been denied shelter	0.90 (0.35–2.32)	1.33 (0.50–3.49)		
Ever denied any services	0.59 (0.22–1.58)	2.30 (0.87–6.11)		
Ever tried to access treatment but unable to	0.87 (0.33–2.27)	1.41 (0.53–3.79)		
Ever left ER without receiving treatment	1.23 (0.40–3.75)	**4.28 (1.42–12.88)**	0.58 (0.16–2.17)	2.42 (0.64–9.18)
**HIV & HCV infection**				
HIV+	0.50 (0.16–1.56)	0.62 (0.20–1.90)		
HCV+	0.71 (0.27–1.90)	0.76 (0.28–2.05)		
**Cultural Connections**				
Traditional language always/often spoken at home	1.76 (0.62–4.99)	2.18 (0.76–6.22)		

*The following variables were excluded due to cell counts less than 5: Education, ever slept outside >3 nights in a row, ever paid for sex work, ever tried to quit drugs, participated in traditional ceremonies, family always/often live by traditional culture.

Among men (Table 5), 73% (n = 98) reported experiencing racism in ≥1 settings. In multivariable analysis, probable post-traumatic stress was marginally associated with experiencing high levels of racism among men; single status and traditional language remained significantly associated with experiencing low levels of racism.

**Table 5:** Bivariate and multivariate multinomial logistic regression analysis of factors associated with racism among men (n = 134).

Characteristic*	UOR (95% CI)		AOR (95% CI)	
Socio-demographic	Low racism	High racism	Low racism	High racism
City, Prince George	1.75 (0.71–4.33)	1.46 (0.52–4.10)		1.72 (0.63–4.74)
Age	0.97 (0.88–1.07)	0.93 (0.83–1.03)		1.00 (0.90–1.11)
Education, high school or more	0.48 (0.16–1.45)	0.94 (0.29–3.08)		
Relationship status, single	**0.36 (0.13–0.97)**	0.55 (0.18–1.71)	**0.32 (0.10–0.92)**	0.48 (0.18–1.97)
**Historical and ongoing colonial trauma**				
Removed from biological parents	2.40 (0.89–6.44)	1.76 (0.58–5.40)		
Childhood sexual abuse ≤15	1.38 (0.48–3.96)	1.59 (0.49–5.18)		
Ever had a child apprehended	1.63 (0.57–4.64)	1.83 (0.57–5.87)		
Ever physically assaulted	1.00 (0.39–2.59)	2.17 (0.67–7.16)		
Ever attempted suicide	1.30 (0.49–3.43)	2.06 (0.68–6.25)		
Ever sexually assaulted	1.36 (0.52–3.57)	2.10 (0.71–6.17)		
Police ever used physical force against you	2.40 (0.89–6.44)	1.76 (0.58–5.40)		
Probable Post-traumatic stress	1.31 (0.53–3.27)	**3.50 (1.16–10.63)**	0.97 (0.36–2.64)	**2.85 (0.88–9.20)**
**Substance Use**				
Ever overdosed	0.93 (0.36–2.42)	1.76 (0.61–5.09)		
Ever injected	0.85 (0.34–2.14)	1.29 (0.44–3.82)		
**Access to services and treatment**				
Ever been denied shelter	1.43 (0.58–3.52)	1.77 (0.62–5.06)		
Ever denied any services	0.81 (0.33–2.01)	2.08 (0.73–5.91)		
Ever tried to access treatment but unable to	1.23 (0.50–3.04)	2.04 (0.69–6.03)		
**HIV & HCV infection**				
HCV+	0.91 (0.36–2.27)	1.53 (0.53–4.39)		
**Cultural Connections**				
Traditional language always/often spoken at home	**4.13 (1.39–12.27)**	**3.01 (0.91–9.10)**	**4.00 (1.28–12.48)**	2.56 (0.73–9.01)
Family always/often live by traditional culture	1.31 (0.44–3.89)	1.42 (0.44–4.57)		

*The following variables were excluded due to cell counts less than 5: identifying as two-spirit, parents in residential school, ever been in detention/prison; ever tried to quit drugs, ever left ER without receiving treatment, and HIV status.

## Discussion

**Fig 1:**
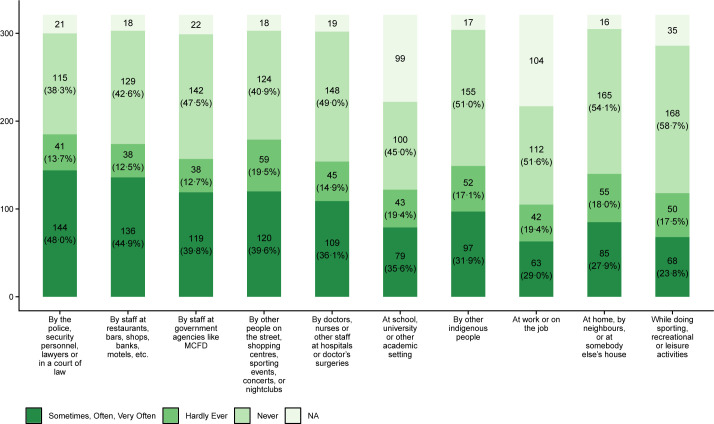
Response distribution of self-reported racism across nine settings (n = 321). Response counts are provided for each category (sometime, often, very often; hardly ever; never; NA). Percentages were calculated after excluding missing values and are shown in brackets.

Indigenous people who use drugs do so to cope with unresolved trauma and ongoing pain [32]. Cedar Project participants who have been in the child welfare system have shared with us the immense stress they endured when they were taken from their families; describing how they ran away from foster homes, suffered from mental health illness, had difficulty building and maintaining relationships, and turned to self-medication to cope with their pain [33]. Nearly 80% of the young Indigenous people who use criminalized drugs in BC involved in this study reported that they had experienced racism. Almost 40% had experienced racism in at least six institutional and public settings (see Fig 1). Participants experienced high levels of racism in private businesses such as banks, restaurants, bars, shops and motels (42%) and public places such as on the street, special events, and shopping centres (37%). The frequent experience of Indigenous-specific racism, combined with the wide variety of settings and contexts where it took place, highlight how racism in BC remains a structural, systemic, and interpersonal issue [24]. Multilevel change is required to transform BC institutions towards anti-racist laws, policies, organizations, and services that support Indigenous health and wellbeing [6, 24]. The following discussion unpacks findings and recommendations related to participants’ experiences with imposed settler institutions in BC: the criminal justice system, the mental health and addictions system, the health system, and the child apprehension system.

### Criminal justice system

Police were the most common source of racism in this study, with 45% of participants reporting experiencing racism in their interactions. This is consistent with previous Cedar research revealing frequent and concerning interactions with police in both Prince George and Vancouver [18]. Implementation of Canada’s racist settler-colonial laws, including systems of incarceration (reserve system, prison system) and child apprehension (residential ‘schools’, child ‘welfare’ system) has been carried out by police forces across Canada [18]. Understanding this historic context is critical to acknowledging how in the present day, Indigenous peoples continue to be subjected to disproportionate discretionary street check and incarceration rates compared to non-Indigenous people [34–36] and experience disturbing disparities in years of life lost to incarceration [37]. Structural and systemic reforms are urgently needed, including an Indigenous-led police oversight body with investigative powers and authority. Racist policing also has profound health and safety implications for people who are racialized and use illicit drugs, especially those experiencing mental health issues [25, 38]. Calls to de-fund/de-task the police represent the life-and-death need to examine current police structures and responsibilities, particularly in the context of mental health and substance use [39].

### Mental health & addictions system

Participants screened for PTSD were nearly three times more likely to experience high interpersonal racism; these associations were particularly strong among women. Previous research underscores the detrimental impact of racism on mental health, leading to higher psychological distress and substance use [14, 40, 41]. Conversely, participants who tried to quit using substances were four times more likely to experience high interpersonal racism compared to those who had not tried to quit. This finding is possibly linked to encounters with health and substance use services where racism is deeply embedded [6]. Mental health and substance use provisions and policies are based on racist ideologies, removing Indigenous peoples’ agency and inflicting harm [42]. This is evident in “wellness checks,” a police response to mental health crisis, which recently led to the fatal shootings of Chantel Moore (Tlaoquiaht Nation) and Rodney Levi (Metepenagiag Nation), who needed help and care [43]. Criminalization of drug use, a policy measure rooted in racism [44], has also created disproportionate harm to Indigenous peoples [10, 45, 46]. During the first COVID-19 pandemic wave, Indigenous people died from overdoses at 5.6 times the rate of other BC residents, with Indigenous women dying at 8.7 times the rate of non-Indigenous women in 2019 [12]. Encounters with child ‘welfare’ likely contribute to the overdose crises experienced by Indigenous families. A recent study highlighted the devastating impact of child apprehension on Indigenous mothers, as they were more likely to experience a non-fatal overdose relative to non-Indigenous women [47]. Indigenous-led wholistic mental wellness and harm reduction services that support self-determination are essential [42]. Indigenous leaders and activists have called for structural interventions, including legislative changes to safe supply access, and reallocation of police funding to housing and mental health initiatives.

### Health system

Thirty-four percent of participants reported they had experienced high levels of racism from health providers, and leaving ER without receiving treatment was associated with three times greater likelihood of experiencing high interpersonal racism. Encounters between Indigenous patients and non-Indigenous healthcare providers continue to be affected by deep-rooted racial bias, stereotypes, and racism that remain unaddressed [4, 48–50]. Resulting negative experiences can include instances when: concerns are discounted, assumptions are made about behaviour, someone is blamed or belittled, witnessing harm to loved ones, cultural health practices are diminished, and/or rights and agency are undermined [2, 6, 8, 51–53]. Impacts on access to health services perpetuate negative consequences of racism on health and wellbeing [6, 54]. This study’s findings affirm that Indigenous-specific racism is a health emergency. It is essential that the BC health system, at all levels, takes immediate and sustained action to implement the *In Plain Sight* recommendations [6].

### Child apprehension system

Racism in interactions with government workers from the MCFD were reported by 37% of participants. Women who had a child apprehended were five times more likely to report experiencing high levels of racism. Cedar participants are deeply impacted by multigenerational state-enforced separation of children from families. More than half of participants had a parent who attended residential ‘schools’, and 65% had been through the foster system [55, 56]. Links between child apprehension and adverse health outcomes demonstrated in Cedar data include increased risk of HIV, HCV, and suicide, as well as reduced likelihood of success in HIV treatment [55, 57–60]. In January 2020, the federal government introduced Bill C-92 to reduce the number of Indigenous young people in state custody, affirm Indigenous jurisdiction over child and family services, establish national principles, and contribute to implementation of United Nations Declaration on the Rights of Indigenous Peoples (UNDRIP) [61]. However, Gitxsan activist Cindy Blackstock cautioned that lack of clarity around funding could render this legislation a ‘paper tiger’ and hinder meaningful implementation [62]. The Canadian Human Rights Tribunal (CHRT) has found Canada guilty of deliberately underfunding on-reserve child ‘welfare’ and ordered the federal government to pay $40,000 to each child apprehended–a decision contested by the Canadian government. Federal and provincial governments must uphold commitments to the Truth and Reconciliation Commission of Canada and UNDRIP by respecting and fully resourcing Indigenous self-determination related to child protection and ending inequity in public services for First Nations families [61, 63–66].

### Language & culture

Having a traditional language spoken at home while growing up was associated with being nearly three times more likely to experience high interpersonal racism. This association was particularly pronounced among men. For many Cedar participants, resilience in the face of adversity is rooted in connections with land, culture, and family [67]. Yet, this study demonstrates that participants who grew up speaking their language are more likely to experience high levels of racism. This relationship has also been documented in research with urban Indigenous Australians–those who most strongly identify with their culture are at greater risk of experiencing racism [30, 68]. Singular focus on enhancing Indigenous resilience to fight oppressive systems without transformative systemic change that challenges the roots of oppression is insufficient. Indigenous self-determination needs to be prioritized across all siloes to transform institutions of risk into institutional wellness and risk environments into support environments.

## Limitations

Participants experience stigma and discrimination resulting from intersecting systems of oppression. Study results likely underestimate the impact of racism. Poor treatment may be attributed to other aspects of participants’ lived experiences and identities. As data are self-reported, behaviours that may be painful to recall, illegal or stigmatizing maybe under-reported. However, Cedar staff work to mitigate self-report bias by building trusted relationships over 18 years of engagement with participants and through repeated assurance of confidentiality. As no sampling frame exists to enumerate the total population of young Indigenous people who have used drugs, certain experiences may be over- or under-estimated. However, our rigorous sampling methods and refreshment of the cohort before administration of the MIRE strengthen our conclusions. Finally, given the cross-sectional nature of this analysis, it is not possible to determine direction or causality of the relationships identified.

## Conclusion

Settler colonialism in Canada is born of and maintained by racism and white supremacy. Findings demonstrate that Cedar participants experience interpersonal racism in everyday encounters with both BC systems and residents. Specifically, Cedar participants suffering from encounters with the child apprehension system, trauma, substance use, and the acute health system (e.g., emergency), were more likely to experience interpersonal racism. When Indigenous self-determination is not respected racism continues to be hard-wired in everyday life, and disparities between Indigenous and non-Indigenous people are maintained and reinforced [51, 53, 69]. To break these oppressive cycles, we must dismantle racism at every level, including structures, systems, services, and interpersonal relationships [24]. B.C. has begun to build a pathway to structural change, *Bill 41*: *Declaration on the Rights of Indigenous Peoples Act*, requiring laws to be brought into alignment with the UNDRIP [64]. However, Indigenous-led mechanisms are needed to hold mainstream systems accountable for harmful racist policies and actions. Finally, at an interpersonal level, white and racialized settlers must examine their relationship to anti-Indigenous racism and position themselves in relation to self-determining Indigenous Nations.
